# Molecular Basis of The Retinal Pigment Epithelial Changes That Characterize The Ocular Lesion in Toxoplasmosis

**DOI:** 10.3390/microorganisms7100405

**Published:** 2019-09-29

**Authors:** Shervi Lie, Bárbara R. Vieira, Sigrid Arruda, Milena Simões, Liam M. Ashander, João M. Furtado, Justine R. Smith

**Affiliations:** 1 College of Medicine & Public Health, Flinders University, Adelaide 5042, Australia; shrvlie@gmail.com (S.L.);; 2 Division of Ophthalmology, Ribeirão Preto Medical School, University of São Paulo, Ribeirão Preto 14049-900, Brazil; barbararvieira@yahoo.com.br (B.R.V.); milenasfsilva@yahoo.com.br (M.S.); furtadojm@gmail.com (J.M.F.)

**Keywords:** ocular toxoplasmosis, retinal pigment epithelium, retinochoroiditis, *Toxoplasma gondii*

## Abstract

When a person becomes infected with *Toxoplasma gondii*, ocular toxoplasmosis is the most common clinical presentation. The medical literature describes retinitis with surrounding hyperpigmentation secondary to proliferative changes in the retinal pigment epithelium, which is sufficiently characteristic that investigation often is not needed to make the diagnosis. We aimed to establish the frequency of “typical” ocular toxoplasmosis and delineate its molecular basis. Among 263 patients presenting consecutively with ocular toxoplasmosis to Ribeirão Preto General Hospital in Brazil, where *T. gondii* infection is endemic, 94.2% of 345 eyes had retinal hyperpigmentation. In ARPE-19 and primary human retinal pigment epithelial cell monolayers exposed to minimal numbers of *T. gondii* tachyzoites, the proliferation marker–KI-67–was increased in uninfected cells, which also were rendered more susceptible to infection. RT-qPCR and ELISA detected increased expression of vascular endothelial growth factor A (VEGF) and insulin-like growth factor (IGF)1, and decreased expression of thrombospondin (TSP)1 by infected cells. Blockade of VEGF and IGF1—or supplementation of TSP1—reversed the proliferation phenotype in uninfected cells. Our findings confirm that hyperpigmentation is a characteristic feature of retinitis in ocular toxoplasmosis, and demonstrate that *T. gondii*-infected human retinal pigment epithelial cells secrete VEGF and IGF1, and reduce production of TSP1, to promote proliferation of adjacent uninfected cells and create this disease-specific appearance.

## 1. Introduction

When a human is infected with the protozoan parasite, *Toxoplasma gondii*, the most common clinical manifestation is an inflammatory eye condition known as ocular toxoplasmosis [1]. Approximately one-third of the world population is infected with *T. gondii* [2], and the prevalence of ocular toxoplasmosis varies from less than 1% to approximately 18% of persons, depending on geographical location [3]. For the majority, ocular toxoplasmosis involves the posterior eye and is characterized by recurrent necrotizing retinitis, with frequent extension of the inflammation and tissue destruction into the choroid, which encapsulates the retina. The toxoplasmic lesion has a central area of tissue destruction, which when the retina and choroid are breached reveals white sclera at the base, and is surrounded by a ring of pigment. Recurrences of retinitis occur at the edge of the pigment ring. This clinical picture is sufficiently characteristic that it is referred to in the ophthalmic medical literature as “typical ocular toxoplasmosis” [4]; most ophthalmologists diagnose the condition and initiate treatment with anti-parasitic and corticosteroid drugs on the basis of the clinical appearance alone, without the need to sample and test ocular fluids [5].

Changes in the pigment patterning of the retina indicate the involvement of the retinal pigment epithelium, which lies deep to the neural retina, directly opposed to the choroid [6]. The multi-functional retinal pigment epithelium is a target cell for *T. gondii*, with actively replicating tachyzoites identified in these cells during active disease [7,8,9]. Historical articles, documenting histopathological findings in human eyes removed from immunocompromised patients with relatively severe ocular toxoplasmosis, make reference to alterations of this cellular layer [1]. In their detailed descriptions, Yeo et al. [10] and Parke et al. [11] report multilaminar placoid proliferation of the retinal pigment epithelium; cells may show pseudo-sarcomatous changes, including pleomorphic nuclei and large nucleoli. More recently, optical coherence tomography has been used to image ocular toxoplasmosis in the living patient, and demonstrate retinal pigment epithelial changes, including thickening and splitting [12]. The report of a study involving intraperitoneal infection of C57BL/6 mice with ME-49 strain *T. gondii* cysts, and examination of the eyes post-mortem by electron microscopy, suggested that migration of retinal pigment epithelial cells within the retina also might contribute to the clinical picture [13].

The characteristic clinical appearance of ocular toxoplasmosis suggests a specific interaction between *T. gondii* and host retinal pigment epithelial cells. To understand the nature of this interaction, we conducted translational bench research using human retinal pigment epithelial cells and naturally occurring *T. gondii* strains. However, first, we sought to quantify the clinical dogma that ocular toxoplasmosis presents with a lesion characterized by retinal hyperpigmentation—in 345 eyes of 263 patients with ocular toxoplasmosis, presenting to an inflammatory eye disease clinic in Brazil, where infection is endemic, this appearance was present in 94.2% of eyes. We show that human retinal epithelial cells secrete growth factors in response to infection with *T. gondii* that promote the proliferation of neighboring uninfected cells, which increases the susceptibility of these cells to infection with the parasite.

## 2. Materials and Methods

### 2.1. Patients

Between April 2015 and July 2017, patients presenting consecutively to the Uveitis Outpatient Clinic at the Ribeirão Preto General Hospital, Ribeirão Preto, São Paulo, Brazil, with ocular toxoplasmosis were enrolled in this study. This tertiary referral clinic serves a population of approximately 1.7 million people in a region where the prevalence of *T. gondii* infection is estimated at 60% [14]. The diagnosis of ocular toxoplasmosis required the presence of one or more foci of retinal inflammation, plus serological testing indicative of infection with *T. gondii* (i.e., serum *T. gondii* immunoglobulin (Ig)M and/or IgG). All patients had a complete ophthalmic examination, including dilated examination of the posterior segment of both eyes; patients were excluded if the posterior segment could not be adequately visualized because of complications that included cataract or persistent vitreous opacity. In addition to *T. gondii* serology, if the clinical presentation suggested a differential diagnosis—including syphilis, tuberculosis, herpes virus infection, cytomegalovirus infection, or sarcoidosis—the relevant testing was performed to exclude these diseases. In selected cases, anterior eye fluid (aqueous) was tested for *T. gondii* DNA by polymerase chain reaction (PCR).

The clinical data that were collected from the patients included: demographics, including gender, age, and self-stated ethnicity; classification of ocular toxoplasmosis; mode of infection, as verified by medical history; results of serological studies; number and location of retinal lesion(s); and the status of retinal pigmentation. For classification of ocular toxoplasmosis, active ocular toxoplasmosis was defined as focal retinal inflammation with retinal thickening and whitening with indistinct border, with a cellular response in the vitreous; active ocular toxoplasmosis was defined as primary if there was no associated retinal scarring and as recurrent if there was an adjacent retinal scar. Inactive ocular toxoplasmosis was defined as focal retinal scarring with atrophy of the neural retina, and with or without atrophy of the choroid. These definitions are consistent with well described clinical observations in this disease [4]. For those patients classified as having active ocular toxoplasmosis, the eyes were examined again at 2–3 and 68 weeks after presentation, unless the clinical situation indicated more frequent review.

### 2.2. Primary Antibodies, Recombinant Proteins, and Enzyme-Linked Immunosorbent Assay Kits

Rabbit polyclonal anti-human antigen KI-67 (KI-67) antibody was sourced from Abcam (Cambridge, U.K.), and goat polyclonal anti-human vascular endothelial growth factor A (VEGF) antibody and goat polyclonal anti-human insulin-like growth factor 1 (IGF1) antibody were sourced from R&D Systems (Minneapolis, MN, USA). Rabbit immunoglobulin (Ig)G and goat IgG were purchased from Merck Sigma-Aldrich (St Louis, MO, USA) and Vector Laboratories (Burlingame, CA, USA), respectively. Human recombinant thrombospondin 1 (TSP1) was sourced from R&D Systems. The human VEGF and TSP1 enzyme-linked immunosorbent assay (ELISA) kits were obtained from R&D Systems (catalogue numbers: DVE00 and DTSP10), and the human IGF1 ELISA kit was obtained from Biovendor (Brno, Czech Republic).

### 2.3. Cells

The human retinal pigment epithelial cells used in these experiments included a commercially available cell line, and primary isolates generated from human cadaveric donor eyes that were obtained from the Eye Bank of South Australia (Adelaide, Australia) within 24 h of death. The ARPE-19 cell line (ATCC, Manassas, VA, USA) is a spontaneously arising retinal pigment epithelial cell line derived from the normal eyes of a 19 year old male [15]. ARPE-19 cells were maintained in 1:1 Dulbecco’s modified Eagle’s medium:F12 medium (DMEM:F12, Thermo Fisher Scientific-GIBCO, Grand Island, NY, USA), supplemented with 10% heat-inactivated fetal bovine serum (FBS, Bovogen Biologicals, Keilor East, Australia, or GE Healthcare-HyClone, Logan, UT, USA) at 37 °C and at 5% CO_2_ in air. The term “senescent ARPE-19 cells” refers to ARPE-19 cells that were cultured to confluence, and after change of medium, were incubated for 7 days without passage.

Primary human retinal pigment epithelial cells were isolated, cultured, and immunophenotyped as we have described previously [16]. In brief, after removal of vitreous and neural retina, the eyecup was treated with 0.5 mg/mL collagenase IA and 0.5 mg/mL collagenase IV (Merck Sigma-Aldrich, St Louis, MO, USA) in Hanks balanced salt solution (HBSS) at 37 °C, and the retinal pigment epithelium was removed by scraping in DMEM:F12 GlutaMAX (Thermo Fisher Scientific-GIBCO) supplemented with 1× insulin-transferrin-selenium (ITS) supplement (Thermo Fisher Scientific-GIBCO), 100 U/mL penicillin-streptomycin (Thermo Fisher Scientific-GIBCO), 1 μg/mL amphotericin (Merck Sigma-Aldrich), and 10% FBS. Cells were seeded on 35 mm diameter collagen IV-coated dishes (Corning, Corning, NY, USA) and incubated at 37 °C and 5% CO_2_ in air. After one week, concentration of the ITS in the supplemented medium was halved. Following initial growth, cells were passaged by trypsinization into 6-well plates (9.6 cm^2^ growth area) and further expanded in DMEM:F12 medium with 0.5× ITS supplement and 5% FBS until confluent, using brief treatments with 0.05% trypsin to remove any contaminating cells. Both ARPE-19 and primary human retinal pigment epithelial cells are contact-inhibited in culture.

### 2.4. Parasites

*T. gondii* GT-1 and DEG strains (gift of Dr. L. David Sibley, PhD, Washington University, St Louis, MI, USA) were maintained in tachyzoite form by serial passage in human neonatal dermal fibroblast monolayers (Thermo Fisher Scientific-Cascade Biologics, Portland, OR, USA) in DMEM supplemented with 40 mM sodium bicarbonate and 1% FBS at 37 °C and 5% CO_2_ in air. For every experiment, *T. gondii* viability was evaluated by plaque assay; viability in this assay was required to be at least 15% or 2% for the virulent GT-1 strain and the avirulent DEG strain, respectively, consistent with published measurements [17,18].

Yellow fluorescent protein (YFP)-expressing GT-1 tachyzoites were generated as previously described [17,19]. In brief, GT-1 tachyzoites were transfected with pTUBYFP-YFPsagCAT, which is a 9289-bp plasmid expressing a tandem-repeat YFP element and chloramphenicol resistance (gift of Dr. Boris Striepen, PhD, University of Pennsylvania, Philadelphia, MA, USA). An 800 µL suspension of 5.0 × 10^7^ freshly egressed GT-1 tachyzoites was electroporated with 1 mg/mL pTUBYFP-YFPsagCAT in transfection reagent (i.e., solution of 120 mM potassium chloride, 0.15 mM calcium chloride, 10 mM dipotassium hydrogen phosphate/potassium dihydrogen phosphate, 25 mM 4-(2-hydroxyethyl)-1-piperazineethanesulfonic acid [HEPES], 2 mM ethylenediaminetetraacetic acid, and 5 mM magnesium chloride) supplemented with 2 mM adenosine 5′-triphosphate and 5 mM glutathione, at 2000 V, 50 µF, and 25 Ω on the ECM 630 Electroporation System (BTX-Harvard Apparatus, Holliston, MA, USA). Tachyzoites were subsequently passaged in fibroblast monolayers in DMEM supplemented with 40 mM sodium bicarbonate, 1% FBS and, following the parasite first passage, 2 µM chloramphenicol (Merck Sigma-Aldrich). Fluorescent tachyzoites were passaged for approximately 1 month in chloramphenicol, and cloned by serial dilution.

### 2.5. Infection Protocols

In one series of experiments—involving either (1) detection of KI-67 in cell monolayers infected with *T. gondii*, with manipulation of culture conditions in some assays, or (2) infection of cells with YFP-expressing *T. gondii*—confluent retinal pigment epithelial cell monolayers in 6-well plates were infected with 100 (equivalent to multiplicity of infection (MOI), 0.0001) or 500 (equivalent to MOI, 0.0005) freshly egressed tachyzoites, or retained in medium alone. Cell monolayers were incubated in cell-appropriate medium with 5% FBS for 7 days without disturbance to allow formation of plaques through replication of individual parasites.

In a second series of experiments—performed for the purposes of: (1) preparing conditioned medium for use during cell culture, (2) tachyzoite growth assays, (3) harvesting cellular RNA for reverse transcription (RT)-PCR assays, and (4) collecting culture supernatant for ELISAs—confluent retinal pigment epithelial cell monolayers in 6-well plates were infected with freshly egressed tachyzoites at MOI of 5, or retained in medium alone. Cell monolayers were incubated in cell-appropriate medium with 5% FBS. After 4 h, monolayers were washed 4 times with Dulbecco’s phosphate-buffered saline (DPBS) (Thermo Fisher Scientific-GIBCO) to remove tachyzoites that had not yet invaded cells. The medium was refreshed, and cell monolayers were incubated for a further 20 h.

### 2.6. Antigen KI-67 Immunolabelling

After 7 days in culture after *T. gondii* infection, cell monolayers were washed 2 times with DPBS, fixed with 4% paraformaldehyde for 5 min, washed again, and blocked with 3% normal goat serum (Vector, Burlingame, CA, USA) and 0.05% Triton X-100 (Merck Sigma-Aldrich) in DPBS (blocking buffer) for 1 h at room temperature. The monolayers were incubated overnight at 4 °C with anti-human KI-67 antibody or rabbit IgG (2 µg/mL) in blocking buffer. Subsequently, cell monolayers were washed 3 times with 0.1% Tween-20 in DPBS (Merck Sigma-Aldrich) for 5 min and incubated with Alexa Fluor 488 or Alexa Fluor 594-conjugated goat anti-rabbit antibody (0.5 µg/mL, Thermo Fisher Scientific-Molecular Probes, Eugene, OR, USA) in blocking buffer for 1 h at room temperature. Cultures were washed 3 times with 0.1% Tween-20 in DPBS and fixed with 4% paraformaldehyde for 5 min. Following fixation, cultures were washed twice with DPBS and counter-stained with 4′,6-diamidino-2-phenylindole (DAPI, Merck Sigma-Aldrich).

Labelling was imaged at 100× magnification on the Olympus IX53 Inverted Microscope (Olympus Corporation, Tokyo, Japan). All plaques in *T. gondii-*infected cell monolayers were photographed. In the uninfected controls or monolayers treated with conditioned medium—which did not contain parasites—photographs were taken of 12 pre-designated fields identified by a grid that was scribed on culture surfaces. The area of KI-67 labelling relative to total cellular area was quantified using ImageJ 1.48v software. Images were converted to black/white and thresholds were adjusted to account for background labelling.

### 2.7. VEGF and IGF1 Blockade Assays and TSP1 Supplementation Assay

For the VEGF and IGF1 blockade assays, medium conditioned by *T. gondii* infection or the ‘no infection’ control medium were incubated with anti-VEGF antibody (5 µg/mL), anti-IGF1 antibody (15 µg/mL), anti-VEGF and anti-IGF1 (20 µg/mL total), or goat IgG antibody at matching concentration for 1 h at 37 °C. For the TSP1 supplementation assay, medium conditioned by *T. gondii* infection or the ‘no infection’ control medium were incubated with or without TSP1 (0.5, 1, or 2 µg/mL). Conditioned medium that had been collected from infected or uninfected ARPE-19 cell cultures was filtered through a 0.2 µm filter that excluded tachyzoites. Senescent ARPE-19 cells in 6-well plates (9.6 cm^2^ growth area) were cultured with the conditioned medium, and blocking antibodies or TSP1, or control antibodies or medium alone, for 24 h, at which time the cultures were harvested for KI-67 immunolabelling.

### 2.8. RNA Extraction and Reverse Transcription

At the end of incubation, cell monolayers were washed 4 times with DPBS, subsequently treated with 0.55 mM RLT buffer with β-mercaptoethanol (Qiagen, Hilden, Germany), and frozen at −80 °C ahead of RNA extraction. Total RNA was extracted using RNeasy Mini Kit (Qiagen) according to the manufacturer’s protocol and including the optional on-column DNase treatment. RNA concentration was determined using a NanoDrop 2000 Spectrophotometer (Thermo Fisher Scientific, Waltham, MA, USA). Reverse transcription was performed using iScript Reverse Transcription Supermix for RT-qPCR (Bio-Rad, Hercules, CA, USA), with 1 µg of RNA template yielding 200 µl of cDNA.

### 2.9. Quantitative Real-Time Polymerase Chain Reaction

Quantitative real-time PCR (qPCR) was performed on the CFX Connect Real-Time PCR Detection System with iQ SYBRGreen Supermix (Bio-Rad), using 2 µl of cDNA template per reaction. Amplification consisted of a pre-cycling hold at 95 °C for 5 min; 40 cycles of denaturation at 95 °C for 30 s, annealing at 60 °C for 30 s, and extension at 72 °C for 30 s; and a post-cycling hold at 72 °C for 5 min. All reactions were performed as technical duplicates. Abundance of transcript relative to two stable reference genes—60S acidic ribosomal protein P0 (RPLP0) and peptidylprolyl isomerase A (PPIA)—was calculated using CFX Manager 3.1 software and expressed as normalized expression. Primer sequences and expected product sizes are provided in Supplementary Table S1. For each primer pair, gel electrophoresis was used to confirm a single amplicon of expected molecular weight, and amplicons were purified and sequenced to ensure authenticity. A minimum amplification efficiency of 85% was required for all primer pairs.

### 2.10. Statistical Analysis

The data were analyzed using IBM SPSS Statistics software (IBM Corp, Armonk, NY, USA). Two-tailed Student’s *t*-test was used to make comparisons between two groups, and one-way analysis of variance (ANOVA) test was used to make comparisons across multiple groups. Statistical significance was defined by a *p*-value calculated at less than 0.05.

### 2.11. Research Ethics and Biosafety

Collection of clinical information from patients with ocular toxoplasmosis for the purposes of research was approved by the Ethics Committee in Human Research at Ribeirão Preto General Hospital (protocol number 46015415.2.0000.5440). The use of human cadaver donor eyes for use in research on ocular toxoplasmosis was approved by the Southern Adelaide Clinical Human Research Ethics Committee (protocol number: 175.13). In vitro research with *T. gondii* was approved by the Flinders University Institutional Biosafety Committee (Microbiological Dealing protocol number 2013-08 and Notifiable Low Risk Dealing protocol number 2013-09).

## 3. Results

### 3.1. Typical Retinal Lesions with Hyperpigmentation Represent the Majority of Ocular Toxoplasmosis

**Figure 1:**
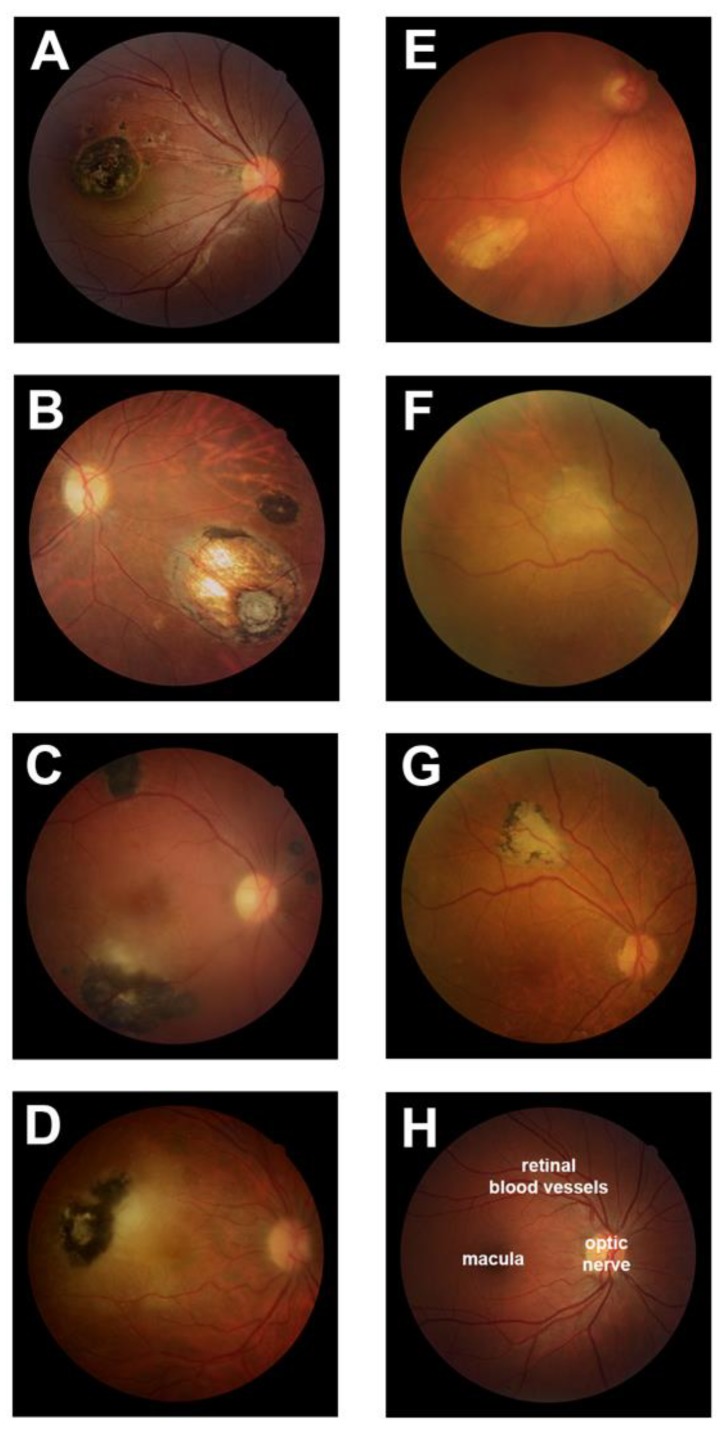
Ocular toxoplasmosis in the group of 263 human subjects, who presented consecutively over a 28 month period to Ribeirão Preto General Hospital, Brazil. Clinical photographs of the posterior segment of the eye in selected individuals with ocular toxoplasmosis. (**A** and **B**) Inactive retinochoroiditis with hyperpigmentation; (**C** and **D**) active retinitis with hyperpigmentation; (**E**) inactive retinochoroiditis without hyperpigmentation; (**F** and **G**) inactive retinochoroiditis that initially presented without hyperpigmentation (**F**), but that became hyperpigmented after an additional 14 months of follow-up (**G**); (**H**) normal posterior eye for comparison, with locations of macula, optic nerve, and retinal blood vessels indicated.

Clinical data were collected on 263 human subjects—139 women and 124 men—who presented consecutively to the Uveitis Service of the Ribeirão Preto General Hospital (Brazil) with ocular toxoplasmosis. The diagnosis was made on clinical examination and results of serological testing as standard, but for six individuals, PCR testing of aqueous for *T. gondii* DNA was required. Amongst this group of patients, the mode of infection was usually unknown (*n* = 226 persons, 85.9%), and consistently, the vast majority had positive *T. gondii* IgG and negative *T. gondii* IgM (*n* = 243 persons, 92.4%). The disease was active in 137 persons (52.1%) and inactive in 126 persons (47.9%). Ocular toxoplasmosis was bilateral in 82 eyes, giving a total of 345 globes with disease. In these eyes, both unifocal and multifocal pathology was observed (*n* = 166 eyes, 48.1% and *n* = 179 eyes, 51.9%, respectively), and lesions were located both in the central retina (*n* = 169 eyes, 49.0%) and peripheral retina (*n* = 221 eyes, 64.1%). The typical hyperpigmented retinal lesion of ocular toxoplasmosis was observed in 325 of the 345 eyes (94.2%), and 246 of the 263 patients (93.5%). In 17 eyes of 14 patients, whose lesions did not have this typical appearance, and who were followed up at a median of 12 months (range = 426 months) beyond the standard period for quiescence, subsequent development of hyperpigmentation was noted in nine eyes. Characteristics of the human subjects and diseased eyes are presented in Table 1 and Figure 1.

### 3.2. Infection with T. gondii Proliferates Human Retinal Pigment Epithelial Cells

**Figure 2:**
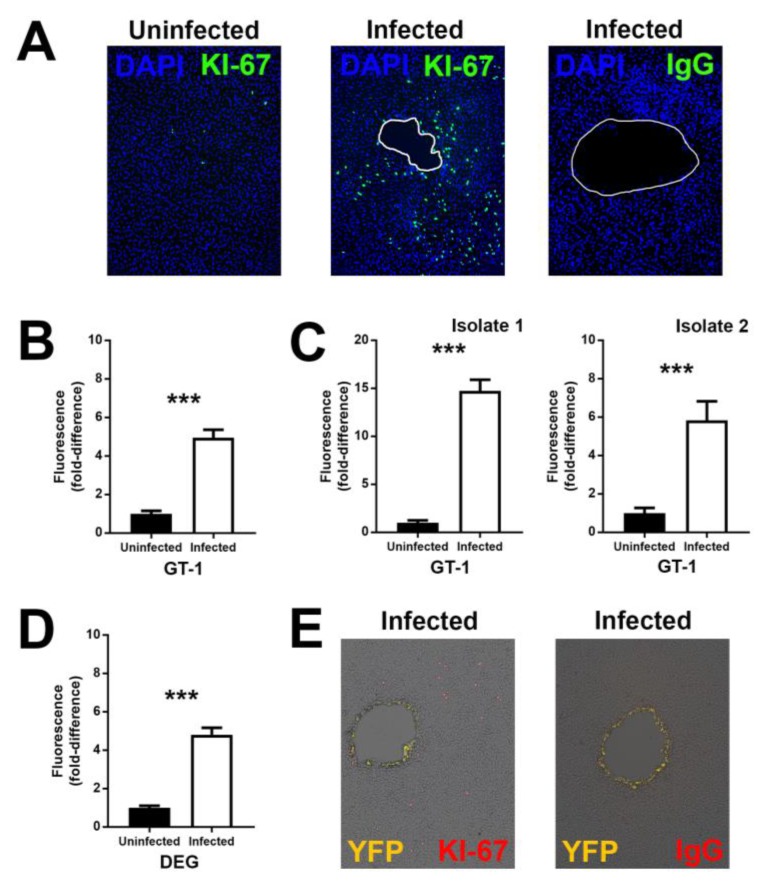
Expression of antigen KI-67 (KI-67) by human retinal pigment epithelial cell monolayers 7 days following infection with *T. gondii* (100 GT-1 strain tachyzoites or 500 DEG strain tachyzoites per 9.6 cm^2^ cell monolayer). (**A**) Representative photomicrographs of ARPE-19 cells infected with GT-1 strain *T. gondii* or maintained uninfected, and immunolabelled for KI-67 or negative control immunoglobulin (IgG). Alexa Fluor 488 (green) with 4′,6-diamidino-2-phenylindole (DAPI) nuclear counterstain (blue). Plaques outlined in white. Original magnification: 100×; (**B**–**D**) graphs of fold-difference in KI-67 immunolabelling, measured in photomicrographs from GT-1-infected versus uninfected (**B**) ARPE-19 cells (representative of three independent experiments) and (**C**) primary cells (isolated separately from two human donors), and (**D**) for DEG-infected versus infected ARPE-19 cells (representative of four independent experiments). Data were analyzed by two-tailed Student’s *t*-test. *n* = up to 32 photomicrographs/condition. Bars represent fold-difference, and error bars indicate standard error of the mean. *** *p* < 0.001. (**E**) Representative photomicrographs of ARPE-19 cells infected with yellow fluorescent protein (YFP)-expressing GT-1 strain *T. gondii*, and immunolabelled for KI-67 or negative control IgG. Alexa Fluor 594 (red) with YFP (yellow). Original magnification: 100×.

The characteristic clinical appearance of ocular toxoplasmosis is associated histopathologically with proliferation of the retinal pigment epithelium [10,11]. To understand the pathogenesis of this clinical phenotype, we first performed experiments using the ARPE-19 human retinal pigment epithelial cell line and GT-1 strain (virulent type I natural isolate) *T. gondii* tachyzoites. Monolayers of epithelial cells were infected with low numbers of tachyzoites (100 parasites per 9.6 cm^2^ monolayer). At 7 days after infection, when infected cell monolayers presented multiple small plaques consistent with growth of individual parasites, expression of the cell proliferation marker–KI-67–was examined by immunocytochemistry. In infected wells, there was a relatively higher number of KI-67-positive cells, remote from the plaques, in comparison to wells that had been grown in parallel, but not infected (Figure 2A). Infected wells stained with negative control antibody showed no positive staining.

Quantification of fluorescence in the cellular area showed a significant increase in infected wells in comparison to uninfected wells (Figure 2B), consistent with a higher number of KI-67-positive cells in infected wells. To address the criticism that a cell line might not faithfully replicate the behavior of primary cells, the same experiment was performed in retinal pigment epithelial cell isolates from human cadaveric donor eye pairs, and this gave the same result (Figure 2C). The experiment was also performed with DEG strain (avirulent type II natural isolate) *T. gondii* tachyzoites (500 parasites per 9.6 cm^2^ monolayer), showing the same outcome (Figure 2D). To confirm that the proliferating cells away from the plaques were not infected with tachyzoites, the same experiment was performed with YFP-expressing GT-1 strain *T. gondii* tachyzoites, verifying that tachyzoites were localized to the margins of the plaque (Figure 2E). In summary, these experiments are consistent with the observation that *T. gondii* tachyzoites induce a proliferation phenotype in uninfected human retinal pigment epithelial cells in the vicinity of an infection.

### 3.3. Infection of Human Retinal Pigment Epithelial Cells with T. gondii Induces Changes in the Levels of Some Growth Factors

**Figure 3:**
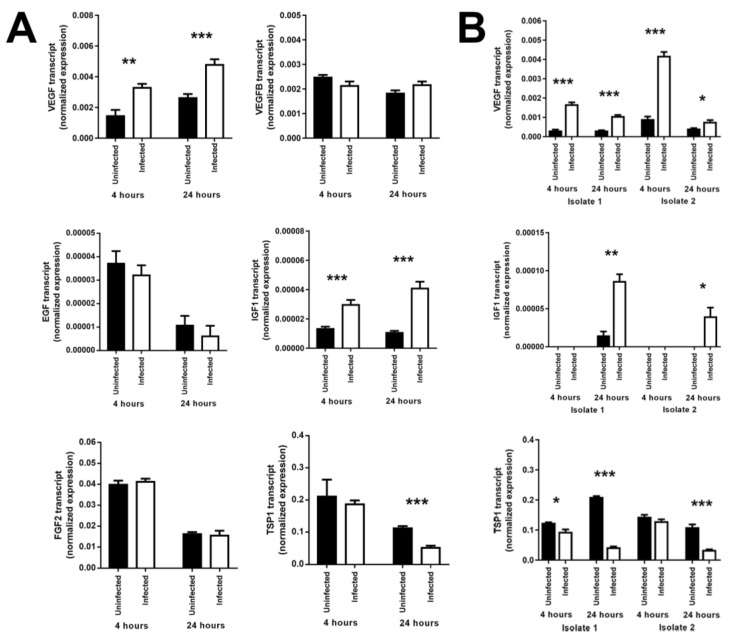
Expression of growth factors by human retinal pigment epithelial cells infected with GT-1 strain *T. gondii* tachyzoites (multiplicity of infection = 5; evaluated time points post-infection = 4 and 24 h). Graphs showing normalized transcript expression for selected growth factors in (**A**) infected versus uninfected ARPE-19 cells (representative of results obtained in at least two of three independent experiments) or (**B**) two infected versus uninfected primary cell isolates. Reference genes were ribosomal protein lateral stalk subunit P0 (RPLP0) and peptidylprolyl isomerase A (PPIA). Data were analyzed by two-tailed Student’s *t*-test. *n* = 6 cultures/condition. Bars represent mean normalized expression, and error bars indicate standard error of the mean. VEGF = vascular endothelial growth factor A; VEGFB = vascular endothelial growth factor B; EGF = epidermal growth factor; IGF1 = insulin-like growth factor 1; FGF2 = fibroblast growth factor 2; TSP1 = thrombospondin 1. * *p* ≤ 0.05, ** *p* ≤ 0.01, *** *p* ≤ 0.001.

In order to identify candidate molecules produced by *T. gondii*-infected human retinal pigment epithelial cells that might account for the induced proliferation of non-infected neighboring cells, we used RT-qPCR to interrogate ARPE-19 cells 4 and 24 h following infection with GT-1 strain tachyzoites for expression of molecules that are produced by and may impact retinal pigment epithelial cell growth: VEGF, vascular endothelial growth factor B (VEGFB), epidermal growth factor (EGF), IGF1, fibroblast growth factor 1 (FGF1), fibroblast growth factor 2 (FGF2), and TSP1 [16,20,21,22,23,24]. In at least two of three independent experiments, we observed significant increases in the expression of VEGF and IGF1 at 4 and 24 h post-infection, and a significant decrease in the expression of TSP1 at 24 h post-infection, comparing infected and uninfected cells. In contrast, infection did not significantly impact cellular expression of EGF, FGF2, and VEGFB, and FGF1 expression was not reliably detected (Figure 3A). To address clinical relevance of the changes in VEGF, IGF1, and TSP1 expression that were observed in the cell line, primary retinal pigment epithelial cell isolates, prepared separately from two human cadaveric donor eye pairs, were similarly infected and interrogated, with consistent results (Figure 3B). These findings suggest that increased production of VEGF and IGF1, and decreased production of TSP1, by infected human retinal pigment epithelial cells might be responsible for the proliferation of adjacent uninfected cells.

### 3.4. Increase in Vascular Endothelial Growth Factor and Insulin-like Growth Factor 1—and Decrease in Thrombospondin 1—by T. gondii-Infected Retinal Pigment Epithelial Cells Promote Proliferation of Uninfected Cells

**Figure 4:**
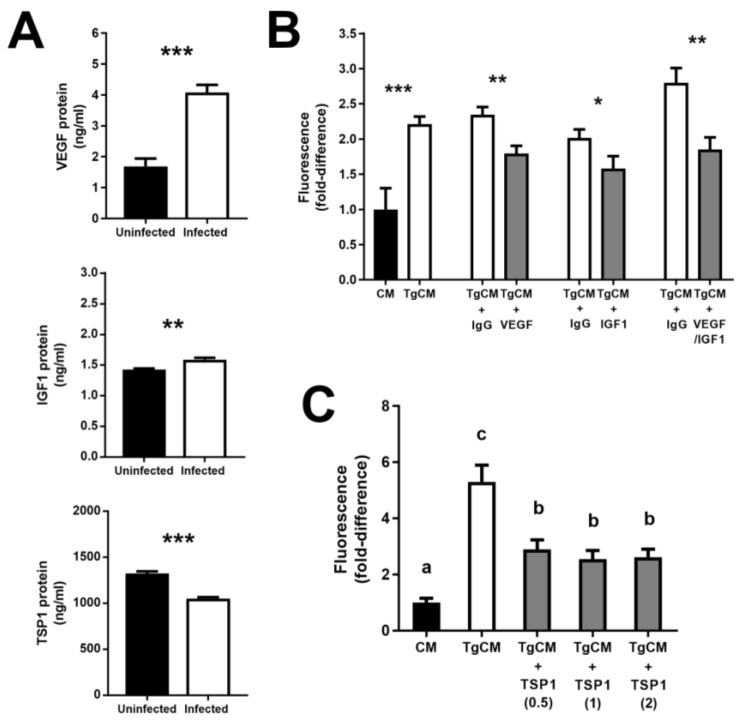
Secretion of growth factors by human retinal pigment epithelial cells infected with GT-1 strain *T. gondii* tachyzoites (multiplicity of infection = 5; evaluated time point post-infection = 24 h), and impact of antibody blockade or protein supplementation on antigen KI-67 (KI-67) expression by conditioned cells. (**A**) Graphs showing protein concentration in supernatant collected from infected versus uninfected ARPE-19 cells, as measured by enzyme-linked immunosorbent assay. Data were analyzed by two-tailed Student’s *t*-test. *n* = 6 cultures/condition. (**B**) Graphs of fold-difference in KI-67 immunolabelling, measured in photomicrographs from ARPE-19 cells exposed for 24 h to conditioned medium collected from *T. gondii*-infected (TgCM) or uninfected (CM) ARPE-19 cells, and (**B**) blocked with antibodies directed against human VEGF (5 µg/mL) and/or IGF1 (15 µg/mL) or negative control immunoglobulin (IgG), or (**C**) supplemented with human TSP1 (0.5–2.0 µg/mL) (representative of two independent experiments). Data were analyzed by (**B**) two-tailed Student’s *t*-test or (**C**) one-way ANOVA. *n* = 12 photomicrographs/condition. Bars represent (**A**) mean secretion or (**B** and **C**) fold-difference, and error bars indicated standard error of the mean. VEGF = vascular endothelial growth factor A; IGF1 = insulin-like growth factor 1; TSP1 = thrombospondin 1. * *p* ≤ 0.05, ** *p* ≤ 0.01, *** *p* ≤ 0.001. In (**C**) different letters denote significant differences.

Given that VEGF, IGF1, and TSP1 produced by *T. gondii*-infected human retinal pigment epithelial cells would have to be acting in a paracrine manner to induce proliferation of uninfected cells, we next sought to confirm secretion of these factors from infected cells. Supernatant collected from ARPE-19 cell cultures 24 h post-infection with GT-1 strain tachyzoites was tested by ELISA. Significant increases and a significant decrease were measured in protein levels of VEGF and IGF1, and TSP1, respectively (Figure 4A).

To evaluate the cell-proliferating potential of VEGF, IGF1, and TSP1 produced by *T. gondii*-infected human retinal pigment epithelial cells, we performed and replicated blockade and supplementation studies. Supernatant was collected from cultures of ARPE-19 cells that had been infected with GT-1 strain tachyzoites 24 h earlier, filtered to remove any extracellular parasites, and applied to uninfected ARPE-19 cell monolayers for 24 h, ahead of quantifying cell proliferation as area of KI-67-positivity. Absence of viable tachyzoites in the filtered, conditioned medium was confirmed by plaque assay. In the presence of anti-human VEGF or IGF1 antibody, induced cell proliferation was significantly reduced in comparison to the control condition, which was incubated with antibody directed against an irrelevant antigen; combining the blocking antibodies resulted in an augmented reduction in the cell-proliferating effect (Figure 4B). In separate experiments, the induced cell proliferation was also significantly reduced by treatment with TSP-1 at a range of concentrations that were based around the level of TSP1 protein secreted by infected cells, as measured by ELISA (Figure 4C). In summary, these experiments show that *T. gondii*-infected human retinal pigment epithelial cells secrete VEGF and IGF1, and reduce the production of TSP1, indicating that these cellular adjustments promote the proliferation of uninfected cells that are growing nearby.

### 3.5. Proliferating Retinal Pigment Epithelial Cells are Relatively Susceptible to Infection with T. gondii Tachyzoites

**Figure 5:**
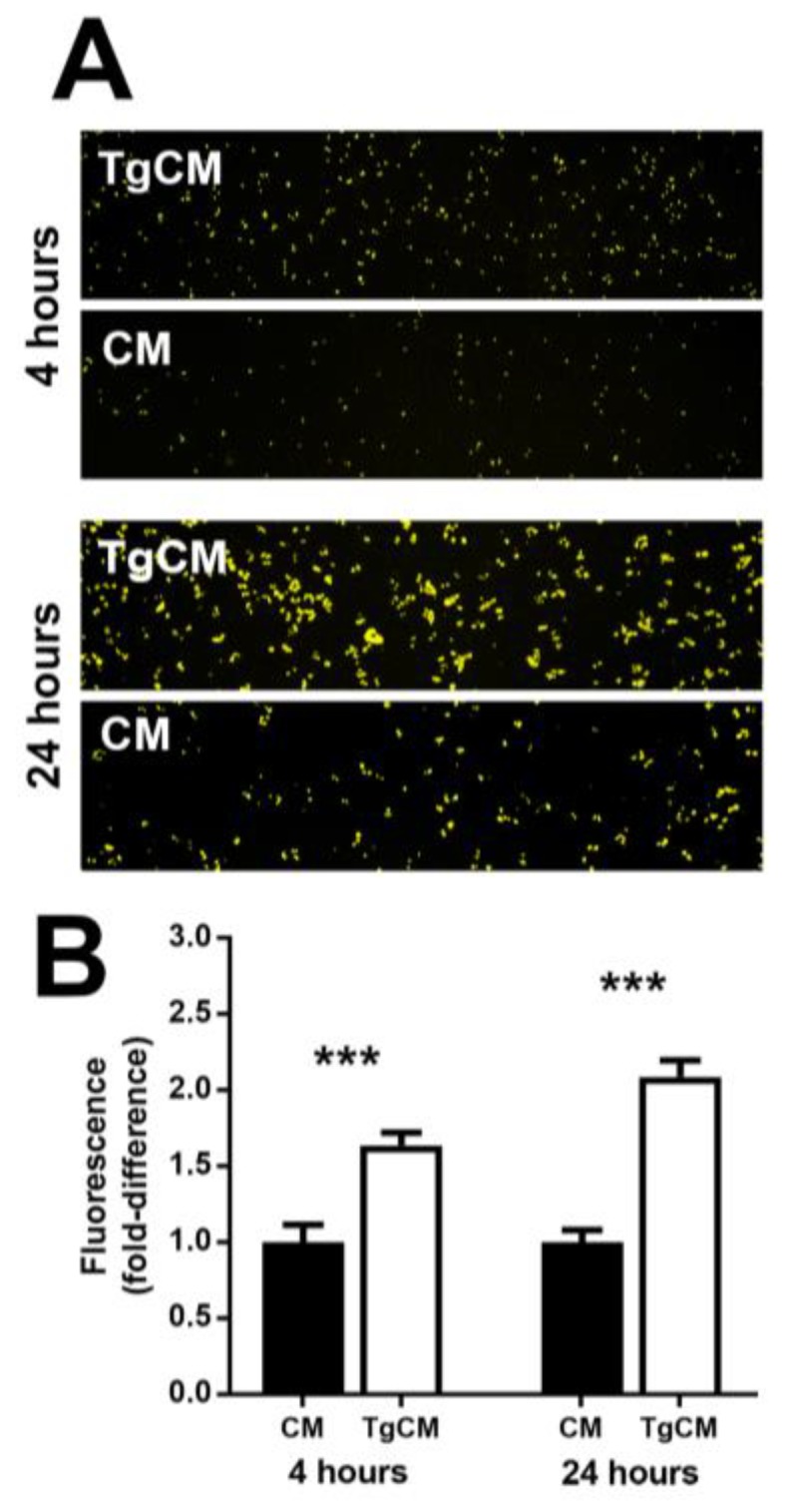
Infection of preconditioned human retinal pigment epithelial cell monolayers with GT-1 strain *T. gondii* tachyzoites (multiplicity of infection = 5; evaluated time points post-infection = 4 and 24 h). (**A**) Representative photomicrographs of ARPE-19 cells 4 and 24 h after infection with yellow fluorescent protein (YFP)-expressing GT-1 strain *T. gondii*, following a 24 h exposure to conditioned medium collected from *T. gondii*-infected (TgCM) or uninfected (CM) ARPE-19 cells. Original magnification: 100×. (**B**) Graph of fold-difference in tachyzoite-associated fluorescence, measured in photomicrographs from YFP-expressing GT-1 strain *T. gondii*-infected ARPE-19 cells (representative of two independent experiments). Data were analyzed by two-tailed Student’s *t*-test. *n* = 12 photomicrographs/condition. Bars represent fold-difference, and error bars indicate standard error of the mean. *** *p* < 0.001.

Because the phase of the cell cycle may impact a cell’s susceptibility to infection with *T. gondii* tachyzoites [25], we examined the impact of infection-induced changes on the susceptibility of human retinal pigment epithelial cells to infection. Supernatant was collected from cultures of ARPE-19 cells that had been either infected with GT-1 strain tachyzoites 24 h earlier or incubated in parallel without infection, filtered to remove any extracellular parasites, and applied to uninfected ARPE-19 cell monolayers, followed by infection with YFP-expressing GT-1 strain tachyzoites. Infection of cells was quantified as relative fluorescence at 4 and 24 h. In replicated experiments, ARPE-19 cells exposed to conditioned medium from infected cultures became significantly more heavily infected than cells exposed to medium from uninfected cultures (Figure 5A,B). These results suggest that the cell-proliferating effect of *T. gondii*-infected human retinal pigment epithelial cells on uninfected cells renders the uninfected cells more susceptible to infection with the parasite.

## 4. Discussion

The retinal lesion with heavy pigmentation, and often a central area of tissue loss, is sufficiently characteristic that ophthalmologists may diagnose ocular toxoplasmosis without need for additional testing. In one of the largest, prospectively recruited cohort of persons with ocular toxoplasmosis described in the literature to date—totaling 263 patients—we show that this picture is seen in the vast majority of patients. We observed the “typical” clinical phenotype in approximately 94% of affected eyes. Among patients without hyperpigmented retinochoroidal scars, who were followed over time post-quiescence, we watched the phenotype subsequently develop in a subset, suggesting our estimate of the typical appearance is conservative. Reports of other large clinical series of ocular toxoplasmosis have not categorized the appearance of the lesion specifically. However, in describing a retrospective review of 233 patients with ocular toxoplasmosis, who were evaluated at the Francis I. Proctor Foundation (San Francisco, CA) between 1977 and 2000, London et al. [26] commented that “atypical presentations” accounted for 6.9% of all cases. In a second retrospective study conducted at University Medical Center Utrecht (Utrecht, The Netherlands) by Bosch-Driessen et al. [27], 24% of 154 patients diagnosed with acute ocular toxoplasmosis between 1990 and 1997 did not have pigmented scars at first presentation, but their retinal lesion “eventually combined with hyperpigmented retinochoroidal scars.”

Seeking to simulate the characteristic ocular lesion in human retinal pigment epithelial cell monolayers, we observed that limited numbers of *T. gondii*-infected cells were capable of inducing a proliferation phenotype—identified by expression of KI-67—in adjacent areas of uninfected cells. We used RT-qPCR to screen infected cells for multiple potential paracrine mediators of this effect, and identified consistent increases in growth factors, VEGF and IGF1, and a consistent decrease in the multifunctional glycoprotein, TSP1. We confirmed parallel changes in the secretion of these factors from infected cells by ELISA. Both VEGF and IGF1 are well-established as being growth factors that promote proliferation of human retinal pigment epithelial cells [20,21,22]. Recent mouse experiments directed at delineating the pathogenesis of age-related macular degeneration have shown that TSP1 deficiency increases proliferation and migratory activity of retinal pigment epithelial cells [24]. Consistently, blockade of VEGF and IGF1, and supplementation of TSP1, significantly reversed the proliferation of uninfected cells exposed to culture supernatant harvested from *T. gondii*-infected cells. These findings suggest that the typical hyperpigmented retinochoroidal lesion of ocular toxoplasmosis is caused, at least in part, by the proliferating action of VEGF and IGF1 secreted by infected cells, along with reduced anti-proliferating action of TSP1, on adjacent uninfected cells.

Biopsy of the retinal pigment epithelium is not indicated or safe in patients with ocular toxoplasmosis, but several groups have investigated the expression of selected molecules in the ocular fluids collected from patients with ocular toxoplasmosis—VEGF, but not IGF1 or TSP1, has been measured. De-la-Torre et al. [28] compared a 27-member “ocular cytokinome” in aqueous fluid collected from Colombian patients with active ocular toxoplasmosis or control patients who had a cataract and no evidence of *T. gondii* infection. Levels of VEGF were significantly increased in the group of patients with ocular toxoplasmosis, and were positively correlated with the number of active lesions and number of scars, plus total number of recurrences. Wiertz et al. [29] and Thieme et al. [30] have reported increased VEGF levels in the aqueous of individual patients with ocular toxoplasmosis. *T. gondii* exists as many strains, which belong to clonal lineages or present atypical or recombinant genetics; these strains are often distinguished on virulence, defined by rates of migration, invasion, and replication in vitro, and lethal dose 100 in vivo in mice [31]. Both virulent and avirulent strains commonly cause human ocular toxoplasmosis [32]. In keeping, we observed the proliferating phenotype in human retinal pigment epithelial cells exposed to virulent (GT-1) or avirulent (DEG) *T. gondii* strains. De-la-Torre et al. [28] compared the effect of *T. gondii* strain on the ocular cytokinome, and observed no difference in the induction of VEGF between strains.

The proliferating phenotype induced in human retinal pigment epithelial cells rendered the cells more susceptible to infection with *T. gondii* tachyzoites. Clearly this phenomenon would promote extension of the infectious focus within the retina. In different host cell populations—including human fibroblasts, trophoblastic cells and hepatoma cells, and mouse macrophages—both pro- and anti-proliferative effects of tachyzoite infection have been reported [25,33,34,35,36]. Molestina et al. [25] have speculated that the “proliferative response” is a mechanism for providing large quantities of nutrient molecules as the parasite seeks to replicate. A proliferating effect of tachyzoite-conditioned medium on adjacent cells has been observed in human fibroblasts, although the host molecules involved were not identified [35]. In this work, we have focused solely on the host cell molecular response, and a key question for future study is whether the changes in human retinal pigment epithelial cell synthesis of VEGF, IGF1, and TSP1, which create this clinical appearance, are induced by the host or the parasite. Tachyzoite molecules may co-opt host cell machinery and effect gene transcription in the host cell [37]. In relation to VEGF specifically, it may be a parasite-derived factor; the research team led by Blader have showed that a *T. gondii*-derived factor triggers activin signaling to activate a key transcription factor for VEGF, hypoxia-inducible factor-1 [38,39]. *T. gondii* is capable of injecting effector proteins into host cells that it does not invade [40]; however, our blockade and supplementation studies used conditioned medium that had been filtered to remove any extracellular parasites and was confirmed to be free of live parasites by plaque assay.

Most of our experiments were performed with ARPE-19 cells, which is a human retinal pigment epithelial cell line that is well characterized [15] and commonly utilized to study mechanisms of intraocular infections, including ocular toxoplasmosis [41,42,43]. Retinal pigment epithelial cells may be isolated from human eyes, but this opportunity is limited by the availability of globes and the limited numbers of cells that can be obtained from individual donors, as well as the tendency for the cells to undergo mesenchymal differentiation in culture [44]. Recently, we have used RNA-sequencing to profile the transcriptome of GT-1 strain *T. gondii*-infected human retinal pigment epithelial cells; as part of this work, we compared the response of ARPE-19 cells and primary human cell isolates [16]. The work showed that, like primary retinal pigment epithelial cells, the ARPE-19 cell line mounts strong immunologic responses, and activation of broadly applicable cell cycle and cell signaling processes, but the molecular pathways involve some different molecules. Thus, in that work, we recommended experimental findings generated with the cell line should be confirmed in primary cells. In the present work, we replicated some experiments with primary cell isolates, including the critical experiment that demonstrated the cell-proliferating phenotype was induced in non-infected cells by tachyzoite-infected cells.

In summary, we have used human retinal pigment epithelial cells and natural isolate strain *T. gondii* to demonstrate the molecular basis of the characteristic retinal hyperpigmentation that is seen in over 90% of patients with toxoplasmic retinitis. Proliferating retinal pigment epithelial cells are relatively susceptible to infection with *T. gondii*, which would promote spread of the parasite within the retina and ultimately increase the likelihood of recurrences of active disease. We speculate that intraocular blockade of VEGF and/or IGF1, and/or supplementation with TSP1, might have a therapeutic application for limiting the extent of the ocular lesion in toxoplasmosis.

## Figures and Tables

**Table 1:** Characteristics of human subjects and eyes with ocular toxoplasmosis (n = 263 individuals and 345 globes). Ig: immunoglobulin.

**Human Subjects, n (%)**	
Gender	
Male	124 (47.1%)
Female	139 (52.9%)
Age (years)	
≤17	29 (11.0%)
18–64	210 (79.8%)
≥65	24 (9.1%)
Self-reported ethnicity	
Multiracial	124 (47.1%)
Caucasian	101 (38.4%)
Afro-Brazilian	35 (13.3%)
Unknown	3 (1.1%)
Form of ocular toxoplasmosis	
Primary active	49 (18.6%)
Recurrent active	88 (33.4%)
Inactive	126 (47.9%)
Mode of infection	
Congenital	24 (9.1%)
Acquired	13 (4.9%)
Unknown	226 (85.9%)
Results of serological studies	
*Toxoplasma gondii* IgG^+^ IgM ^+^	20 (7.6%)
*T. gondii* IgG^+^ IgM^-^	243 (92.4%)
	
**Eyes, n (%)**	
Number of ocular lesion(s)	
1	166 (48.1%)
2–4	147 (42.6%)
≥5	32 (9.2%)
Location of retinal lesion(s)	
Posterior pole	124 (35.9%)
Periphery	176 (51.0%)
Posterior pole and periphery	45 (13.0%)
Hyperpigmented scars	
Present	325 (94.2%)
Absent	20 (5.8%)

## Supplementary Materials

The following are available online at https://www.mdpi.com/2076-2607/7/10/405/s1.

## References

[1] Butler N.J., Furtado J.M., Winthrop K.L., Smith J.R. (2013). Ocular toxoplasmosis II: Clinical features, pathology and management. Clin. Exp. Ophthalmol..

[2] Montoya J.G., Liesenfeld O. (2004). Toxoplasmosis. Lancet.

[3] Furtado J.M., Winthrop K.L., Butler N.J., Smith J.R. (2013). Ocular toxoplasmosis I: Parasitology, epidemiology and public health. Clin. Exp. Ophthalmol..

[4] Holland G.N. (2004). Ocular toxoplasmosis: A global reassessment. Part II: Disease manifestations and management. Am. J. Ophthalmol..

[5] Ozgonul C., Besirli C.G. (2017). Recent developments in the diagnosis and treatment of ocular toxoplasmosis. Ophthalmic Res..

[6] Strauss O., Kolb H., Fernandez E., Nelson R. (2011). The retinal pigment epithelium. Webvision: The Organization of the Retina and Visual System.

[7] Nicholson D.H., Wolchok E.B. (1976). Ocular toxoplasmosis in an adult receiving long-term corticosteroid therapy. Arch. Ophthalmol..

[8] Holland G.N., Engstrom R.E. Jr., Glasgow B.J., Berger B.B., Daniels S.A., Sidikaro Y., Harmon J.A., Fischer D.H., Boyer D.S., Rao N.A. (1988). Ocular toxoplasmosis in patients with the acquired immunodeficiency syndrome. Am. J. Ophthalmol..

[9] Grossniklaus H.E., Specht C.S., Allaire G., Leavitt J.A. (1990). *Toxoplasma gondii* retinochoroiditis and optic neuritis in acquired immune deficiency syndrome. Report of a case. Ophthalmology.

[10] Yeo J.H., Jakobiec F.A., Iwamoto T., Richard G., Kreissig I. (1983). Opportunistic toxoplasmic retinochoroiditis following chemotherapy for systemic lymphoma. A light and electron microscopic study. Ophthalmology.

[11] Parke D.W., Font R.L. (1986). Diffuse toxoplasmic retinochoroiditis in a patient with AIDS. Arch. Ophthalmol..

[12] Orefice J.L., Costa R.A., Scott I.U., Calucci D., Orefice F., Grupo Mineiro de Pesquisa em Doenças Oculares Inflamatórias (MINAS) (2013). Spectral optical coherence tomography findings in patients with ocular toxoplasmosis and active satellite lesions (MINAS Report 1). Acta Ophthalmol..

[13] Tedesco R.C., Smith R.L., Corte-Real S., Calabrese K.S. (2004). Ocular toxoplasmosis: The role of retinal pigment epithelium migration in infection. Parasitol. Res..

[14] Passos A.D.C., Bollela V.R., Furtado J.M.F., Lucena M.M., Bellissimo-Rodrigues F., Paula J.S., Melo L.V.L., Rodrigues M.L.V. (2018). Prevalence and risk factors of toxoplasmosis among adults in a small Brazilian city. Rev. Soc. Bras. Med. Trop..

[15] Dunn K.C., Aotaki-Keen A.E., Putkey F.R., Hjelmeland L.M. (1996). ARPE-19, a human retinal pigment epithelial cell line with differentiated properties. Exp. Eye Res..

[16] Lie S., Rochet E., Segerdell E., Ma Y., Ashander L.M., Shadforth A.M.A., Blenkinsop T.A., Michael M.Z., Appukuttan B., Wilmot B. (2019). Immunological molecular responses of human retinal pigment epithelial cells to infection with *Toxoplasma gondii*. Front. Immunol..

[17] Furtado J.M., Bharadwaj A.S., Ashander L.M., Olivas A., Smith J.R. (2012). Migration of *Toxoplasma gondii*-infected dendritic cells across human retinal vascular endothelium. Invest. Ophthalmol. Vis. Sci..

[18] Khan A., Behnke M.S., Dunay I.R., White M.W., Sibley L.D. (2009). Phenotypic and gene expression changes among clonal type I strains of *Toxoplasma gondii*. Eukaryot. Cell.

[19] Gubbels M.J., Li C., Striepen B. (2003). High-throughput growth assay for *Toxoplasma gondii* using yellow fluorescent protein. Antimicrob. Agents Chemother..

[20] Takagi H., Yoshimura N., Tanihara H., Honda Y. (1994). Insulin-like growth factor-related genes, receptors, and binding proteins in cultured human retinal pigment epithelial cells. Invest. Ophthalmol. Vis. Sci..

[21] Guerrin M., Moukadiri H., Chollet P., Moro F., Dutt K., Malecaze F., Plouet J. (1995). Vasculotropin/vascular endothelial growth factor is an autocrine growth factor for human retinal pigment epithelial cells cultured in vitro. J. Cell. Physiol..

[22] Schlingemann R.O. (2004). Role of growth factors and the wound healing response in age-related macular degeneration. Graefes Arch. Clin. Exp. Ophthalmol..

[23] Leschey K.H., Hackett S.F., Singer J.H., Campochiaro P.A. (1990). Growth factor responsiveness of human retinal pigment epithelial cells. Invest. Ophthalmol. Vis. Sci..

[24] Farnoodian M., Kinter J.B., Yadranji Aghdam S., Zaitoun I., Sorenson C.M., Sheibani N. (2015). Expression of pigment epithelium-derived factor and thrombospondin-1 regulate proliferation and migration of retinal pigment epithelial cells. Physiol. Rep..

[25] Molestina R.E., El-Guendy N., Sinai A.P. (2008). Infection with *Toxoplasma gondii* results in dysregulation of the host cell cycle. Cell. Microbiol..

[26] London N.J., Hovakimyan A., Cubillan L.D., Siverio C.D., Cunningham E.T. (2011). Prevalence, clinical characteristics, and causes of vision loss in patients with ocular toxoplasmosis. Eur. J. Ophthalmol..

[27] Bosch-Driessen L.E., Berendschot T.T., Ongkosuwito J.V., Rothova A. (2002). Ocular toxoplasmosis: Clinical features and prognosis of 154 patients. Ophthalmology.

[28] de-la-Torre A., Pfaff A.W., Grigg M.E., Villard O., Candolfi E., Gomez-Marin J.E. (2014). Ocular cytokinome is linked to clinical characteristics in ocular toxoplasmosis. Cytokine.

[29] Wiertz K., De Visser L., Rijkers G., De Groot-Mijnes J., Los L., Rothova A. (2010). Intraocular and serum levels of vascular endothelial growth factor in acute retinal necrosis and ocular toxoplasmosis. Retina.

[30] Thieme C., Schlickeiser S., Metzner S., Dames C., Pleyer U. (2019). Immune mediator profile in aqueous humor differs in patients with primary acquired ocular toxoplasmosis and recurrent acute ocular toxoplasmosis. Mediators Inflamm..

[31] Behnke M.S., Dubey J.P., Sibley L.D. (2016). Genetic mapping of pathogenesis determinants in *Toxoplasma gondii*. Annu. Rev. Microbiol..

[32] Xia J., Cheng X.Y., Wang X.J., Peng H.J. (2017). Association between *Toxoplasma gondii* types and outcomes of human infection: A meta-analysis. Acta Microbiol. Immunol. Hung..

[33] Brunet J., Pfaff A.W., Abidi A., Unoki M., Nakamura Y., Guinard M., Klein J.P., Candolfi E., Mousli M. (2008). *Toxoplasma gondii* exploits UHRF1 and induces host cell cycle arrest at G2 to enable its proliferation. Cell. Microbiol..

[34] Angeloni M.B., Silva N.M., Castro A.S., Gomes A.O., Silva D.A., Mineo J.R., Ferro E.A. (2009). Apoptosis and S phase of the cell cycle in BeWo trophoblastic and HeLa cells are differentially modulated by *Toxoplasma gondii* strain types. Placenta.

[35] Lavine M.D., Arrizabalaga G. (2009). Induction of mitotic S-phase of host and neighboring cells by *Toxoplasma gondii* enhances parasite invasion. Mol. Biochem. Parasitol..

[36] Wang G., Gao M. (2016). Influence of *Toxoplasma gondii* on in vitro proliferation and apoptosis of hepatoma carcinoma H7402 cell. Asian Pac. J. Trop. Med..

[37] Hakimi M.A., Bougdour A. (2015). Toxoplasma’s ways of manipulating the host transcriptome via secreted effectors. Curr. Opin. Microbiol..

[38] Spear W., Chan D., Coppens I., Johnson R.S., Giaccia A., Blader I.J. (2006). The host cell transcription factor hypoxia-inducible factor 1 is required for *Toxoplasma gondii* growth and survival at physiological oxygen levels. Cell. Microbiol..

[39] Lis A., Wiley M., Vaughan J., Gray P.C., Blader I.J. (2019). The activin receptor, Activin-Like Kinase 4, mediates *Toxoplasma gondii* activation of hypoxia inducible factor-1. Front. Cell. Infect. Microbiol..

[40] Koshy A.A., Dietrich H.K., Christian D.A., Melehani J.H., Shastri A.J., Hunter C.A., Boothroyd J.C. (2012). *Toxoplasma* co-opts host cells it does not invade. PLoS Pathog..

[41] Naemat A., Elsheikha H.M., Boitor R.A., Notingher I. (2016). Tracing amino acid exchange during host-pathogen interaction by combined stable-isotope time-resolved Raman spectral imaging. Sci. Rep..

[42] Nogueira A.R., Leve F., Morgado-Diaz J., Tedesco R.C., Pereira M.C. (2016). Effect of *Toxoplasma gondii* infection on the junctional complex of retinal pigment epithelial cells. Parasitology.

[43] Song H.B., Jun H.O., Kim J.H., Lee Y.H., Choi M.H., Kim J.H. (2017). Disruption of outer blood-retinal barrier by Toxoplasma gondii-infected monocytes is mediated by paracrinely activated FAK signaling. PLoS ONE.

[44] Grisanti S., Guidry C. (1995). Transdifferentiation of retinal pigment epithelial cells from epithelial to mesenchymal phenotype. Invest. Ophthalmol. Vis. Sci..

