# Supplementary material for: Molecular Basis of The Retinal Pigment Epithelial Changes That Characterize The Ocular Lesion in Toxoplasmosis
Source: Microorganisms. 2019 Sep 29;7(10):405. doi: 10.3390/microorganisms7100405 (PMC6843916; doi:10.3390/microorganisms7100405)
Supplement: Supplementary file 1 [file microorganisms-07-00405-s001.pdf]

Supplementary Table 1. Primer sequences, product sizes and NCBI accession numbers for gene transcripts.

**Gene transcript***	**Primer sequences**	**Size (bp)**	**NCBI accession number**
EGF	Forward: 5' GGGAAGATGACCACCACTATTC 3'	349	NM_001963.4
	Reverse: 5' TCTCTGCTCGACTCCTCATAA 3'		
FGF1	Forward: 5' TGAGCGAGTGTGGAGAGAGGTA 3'	114	NM_000800.4
	Reverse: 5' GCTGTGAAGGTGGTGATTTCC 3'		
FGF2	Forward: 5' CAAGCAGAAGAGAGAGGAGTTG 3'	274	NM_002006.4
	Reverse: 5' GCTCTTAGCAGACATTGGAAGA 3'		
IGF1	Forward: 5' GAAGATGCACACCATGTCCT 3'	217	NM_001111283.1
	Reverse: 5' CTCATCCACGATGCCTGTC 3'		
PPIA	Forward: 5'GAGCACTGGAGAGAAAGGATTT3'	355	NM_021130.3
	Reverse: 5'GGTGATCTTCTTGCTGGTCTT3'		
RPLP0	Forward: 5'GCAGCATCTACAACCCTGAA3'	235	NM_053275.3
	Reverse: 5'GCAGATGGATCAGCCAAGAA3'		
TSP1	Forward: 5' AAAGGATAATTGCCCCAACC 3'	177	NM_003246.3
	Reverse: 5' CGGTCTCCCACATCATCTCT 3'		
VEGF	Forward: 5'TGCTGTCTTGGGTGCATT3'	365	NM_001171623.1
	Reverse: 5'GTGCTGTAGGAAGCTCATCTC3'		
VEGFB	Forward: 5'GAGGAAAGTGGTGTCATGGATA3'	273	NM_001243733.1
	Reverse: 5'TCACACTGGCTGTGTTCTTC3'		

*Abbreviations: EGF=epidermal growth factor; FGF1= fibroblast growth factor 1; FGF2= fibroblast growth factor 2; IGF1= insulin-like growth factor 1; PPIA= peptidylprolyl isomerase A; RPLP0= ribosomal protein lateral stalk subunit P0; TSP1= thrombospondin 1; VEGF= vascular endothelial growth factor A; VEGFB= vascular endothelial growth factor B.
